# A novel nomogram to predict perioperative acute kidney injury following isolated coronary artery bypass grafting surgery with impaired left ventricular ejection fraction

**DOI:** 10.1186/s12872-020-01799-1

**Published:** 2020-12-10

**Authors:** Hongyuan Lin, Jianfeng Hou, Hanwei Tang, Kai Chen, Hansong Sun, Zhe Zheng, Shengshou Hu

**Affiliations:** Cardiac Surgery Centre, Fuwai Hospital, Chinese Academy of Medical Sciences and Peking Union Medical College, No. 167, North Lishi street, Xicheng District, Beijing, 100037 China

**Keywords:** AKI, Nomogram, Heart failure, CABG, Prediction model

## Abstract

**Background and objective:**

Heart failure (HF) is a global health issue, and coronary artery bypass graft (CABG) is one of the most effective surgical treatments for HF with coronary artery disease. Unfortunately, the incidence of postoperative acute kidney injury (AKI) is high in HF patients following CABG, and there are few tools to predict AKI after CABG surgery for such patients. The aim of this study is to establish a nomogram to predict the incidence of AKI after CABG in patients with impaired left ventricular ejection fraction (LVEF).

**Methods:**

From 2012 to 2017, Clinical information of 1208 consecutive patients who had LVEF< 50% and underwent isolated CABG was collected to establish a derivation cohort. A novel nomogram was developed using the logistic regression model to predict postoperative AKI among these patients. According to the same inclusion criteria and the same period, we extracted the data of patients from 6 other large cardiac centers in China (*n* = 540) from the China Heart Failure Surgery Registry (China-HFSR) database for external validation of the new model. The nomogram was compared with 3 other available models predicting renal failure after cardiac surgery in terms of calibration, discrimination and net benefit.

**Results:**

In the derivation cohort (*n* = 1208), 90 (7.45%) patients were diagnosed with postoperative AKI. The nomogram included 7 independent risk factors: female, increased preoperative creatinine(> 2 mg/dL), LVEF< 35%, previous myocardial infarction (MI), hypertension, cardiopulmonary bypass(CPB) used and perioperative blood transfusion. The area under the receiver operating characteristic curve (AUC) was 0.738, higher than the other 3 models. By comparing calibration curves and decision curve analyses (DCA) with other models, the novel nomogram showed better calibration and greater net benefit. Among the 540 patients in the validation cohort, 104 (19.3%) had postoperative AKI, and the novel nomogram performed better with respect to calibration, discrimination and net benefit.

**Conclusions:**

The novel nomogram is a reliable model to predict postoperative AKI following isolated CABG for patients with impaired LVEF.

## Background

Coronary artery disease (CAD) is the most common cause of heart failure (HF) [1]. Coronary artery bypass grafting (CABG) surgery is invariably recommended for CAD related HF [2]. Impaired LVEF is supposed as a precursor of HF and indicates a poorer prognosis of HF. Additionally, due to difficulty of the surgery and complexity of the perioperative management, some severe complications of CABG in patients with lower LVEF are concerned [3]. Among these complications, acute kidney injury (AKI) has a higher incidence of 12–48% [4–6]. Consequently, cardiac dysfunction combined with AKI, also known as postoperative multi-organ dysfunction, is assuredly closely associated with mortality or decreased quality of life [7]. Recently, a number of predictive scoring models for AKI have been established. Some of them are extensively utilized, for instance, Cleveland score [8], Mehta score [9] and Simplified Renal Index (SRI) score [10]. However, these models were designed primarily for all cardiac procedures and were based on old clinical data which was collected decades ago, mainly from western populations. As a result, these models might theoretically present poor performance among patients with impaired LVEF. It is of great clinical importance to establish a risk assessment model for such patients. As a reliable and simplified scoring system, the nomogram predictive model could accurately predict postoperative complications, so as to help in risk control, aiming at reducing the morbidity and mortality. We performed the study to identify independent risk factors for postoperative AKI and develop a nomogram backed by external validation for better clinical evaluation.

## Method

From January 2012 to December 2017, all patients (*n* = 1208) who had an impaired LVEF value (LVEF< 50%, measured by echocardiogram) and underwent a isolated CABG procedure at Fuwai Hospital were enrolled as the derivation cohort. Except for those who were allergic to contrast media or had a relative high SCr(> 2 mg/dL) with an estimated glomerular filtration rate (eGFR) less than 30–45 ml/min/1.73m^2^, a contrast-enhanced computerized tomography (CT) scan for aorta was routinely conducted before surgery to screen concomitant aortic anomalies needing surgical correction in the derivation cohort. Detailed information of demographics and perioperative risk factors was collected (Table 1). Based on the KDIGO Clinical Practice Guideline [11] for Acute Kidney Injury, AKI was defined as any of the following: increase in serum creatinine (SCr) ≥0.3 mg/dL within 48 h or increase in SCr ≥1.5 times baseline in 7 days or urine volume < 0.5 mL/kg/hour for 6 h.

**Table 1:** Demographics and risk factors of derivation and validation cohorts

Risk factors	Definition	Derivation cohort(*n* = 1208) N (%)	Validation cohort (*n* = 540) N (%)	P value
Age > 65 years	Older than 65 years	347 (28.7)	200 (37)	< 0.001
Female		174 (14.4)	83 (15.4)	0.65
SCr > 2 mg/dl	Serum creatinine measured before surgery > 2 mg/dl	13 (1.1)	6 (1.1)	1
Chronic kidney disease	Documented past history or fulfilled the criteria of KDIGO 2012	5 (0.4)	10 (1.9)	0.006
Extracardiac arteriopathy	Any one or more of the following: claudication, carotid occlusion or > 50% stenosis, previous or planned intervention on the abdominal aorta, and limb arteries or carotids	98 (8.1)	14 (2.6)	< 0.001
Cerebrovascular accident	Documented past history of coma≥24 h or central nervous system dysfunction≥72 h	42 (3.5)	41 (7.6)	0.003
History of smoking	Prior history of smoking, regardless of whether the patients quit smoking	988 (81.8)	276 (51.1)	< 0.001
Previous cardiac surgery	One or more previous major cardiac operation involving opening the pericardium	45 (3.7)	2 (0.4)	< 0.001
COPD	Long-term use of bronchodilators or steroids for lung disease	6 (0.5)	8 (1.5)	0.07
Diabetes mellitus	Documented past history or fulfilled the criteria of WHO 1999	324 (26.8)	218 (40.4)	< 0.001
NYHA class III or IV	NYHA classification	624 (51.7)	237 (43.9)	0.003
CCS angina class = 4	CCS class 4 angina (inability to perform any activity without angina or angina at rest)	126 (10.4)	7 (1.3)	< 0.001
LVEF< 35%	Assessed by echocardiography (measured before surgery)	85 (7)	45 (8.3)	0.39
History of myocardial infarction	Documented history or ECG evidence including recent myocardial infarction (< 21 days)	564 (46.7)	278 (51.5)	0.07
Previous PCI	Documented history	102 (8.4)	78 (14.4)	< 0.001
Non-elective surgery	Not routine admission for operation	62 (5.1)	11 (2.1)	0.004
Hypertension	Documented past history or SBP > 140 mmHg and/or DBP > 90 mmHg	649 (53.7)	308 (57)	0.217
On-pump surgery	With extracorporeal circulation	648 (53.6)	70 (13)	< 0.001
Perioperative IABP	Intra-aortic balloon pump used before or after surgery	14 (1.2)	82 (15.2)	< 0.001
Ventilation time > 24 h	Ventilation time longer than 24 h	249 (20.6)	94 (17.4)	0.1352
Perioperative transfusion	Transfusion before or after surgery	140 (11.6)	154 (28.5)	< 0.001
AKI	Fulfilled the criteria of KDIGO 2012	90 (7.5)	104 (19.3)	< 0.001

*AKI* acute kidney injury, *CCS* Canadian cardiovascular society, *COPD* chronic obstructive pulmonary disease, *DBP* diastolic blood pressure, *ECG* electrocardiography, *LVEF* left ventricular ejection fraction, *NYHA* New York heart association, *PCI* percutaneous coronary intervention, *SBP* systolic blood pressure, *WHO* world health organization

**Fig. 1:**
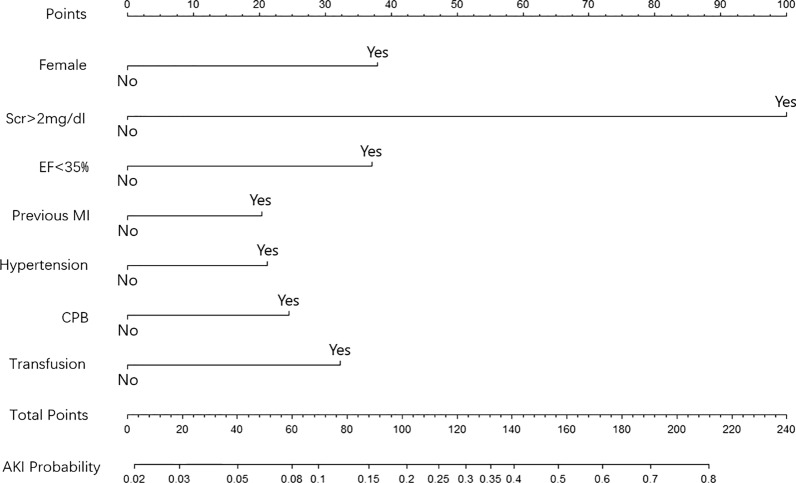
The novel nomogram score system

In the derivation cohort (*n* = 1208), all the possible risk factors were screened by univariate analyses and a logistic regression was performed to select the independent risk factors as predictors. Subsequently, we constructed a nomogram model (Fig. 1) which could predict the probability of postoperative AKI for each individual. By assigning a risk score for each predictor based on its regression coefficient, the nomogram could provide a probability corresponding to the sum of all predictors’ risk scores. The calibration plot was drawn to evaluate the calibration of the model. The AUC and DCA were used to evaluate the discrimination and clinical net benefit of the model. The calibration, discrimination and clinical net benefit of the novel model were compared with the other three prediction models (Cleveland score, Mehta score and SRI score).

We used a data set of 540 patients extracted from the China-HFSR database to externally validate this model. We enrolled patients with impaired LVEFs undergoing isolated CABG during the same period as the derivation cohort. The calibration was evaluated by the calibration plot. AUC and DCA were used to evaluate the discrimination and clinical net benefit.

This study had been approved by institutional review board of Fuwai hospital, Peking union medical college and Chinese academy of medical sciences.

### Statistical analysis

All the variables were categorical and presented as frequencies (percentages). For univariate analyses, differences between groups were assessed using chi-square test or Fisher exact test. Factors with *P* value larger than 0.1 were excluded from the multivariate analysis. A multivariable logistic regression was performed using ‘Enter’ method to construct a nomogram. As mentioned above, calibration was performed using a calibration plot, discrimination was assessed by AUC and DCA was used to evaluate the clinical net benefit. All analyses were performed using the R software, version 3.5, and the SPSS, version 20.0. A two-sided *P* < 0.05 indicated statistical significance.

## Results

The derivation cohort consisted of 1208 patients with a median age of 61 (ranged from 22 to 84) and 174/1208 (14.4%) were female. The validation cohort consisted of 540 patients with a median age of 62 (ranged from 34 to 85) and 83/540 (15.3%) were female. The rate of AKI was 7.5% (90/1208) in the derivation cohort and 19.3% (104/540) in the validation cohort. Demographics and perioperative characteristics of the derivation and validation cohorts were listed in Table 1.

In multivariate analysis, female, SCr > 2 mg/dl, LVEF< 35%, previous MI, hypertension, CPB used and perioperative transfusion were significant predictors for postoperative AKI. All the coefficients and other parameters of the multivariate logistic regression were listed in Table 2. The novel nomogram was developed based on logistic regression (Fig. 1).

**Table 2:** Coefficents of variables in multivariate logistic regression (the model intercept = −3.9273)

Risk factor	P value	β coefficient	S.E	Wald value
Age > 65y	0.23	0.2618	0.2183	1.199
Female	0.005	0.6962	0.2464	2.826
Scr > 2 mg/dl	< 0.001	2.2135	0.5728	3.865
CKD	0.084	1.6619	0.9613	1.729
LVEF< 35%	0.004	0.9189	0.3162	2.906
Previous MI	0.027	0.4528	0.2045	2.215
Hypertension	0.02	0.4955	0.2133	2.323
CPB	0.012	0.5348	0.214	2.5
IABP	0.379	0.5879	0.6684	0.879
Transfusion	0.017	0.626	0.2623	2.387
Ventilation> 24 h	0.064	0.4229	0.2284	1.851

**Fig. 2:**
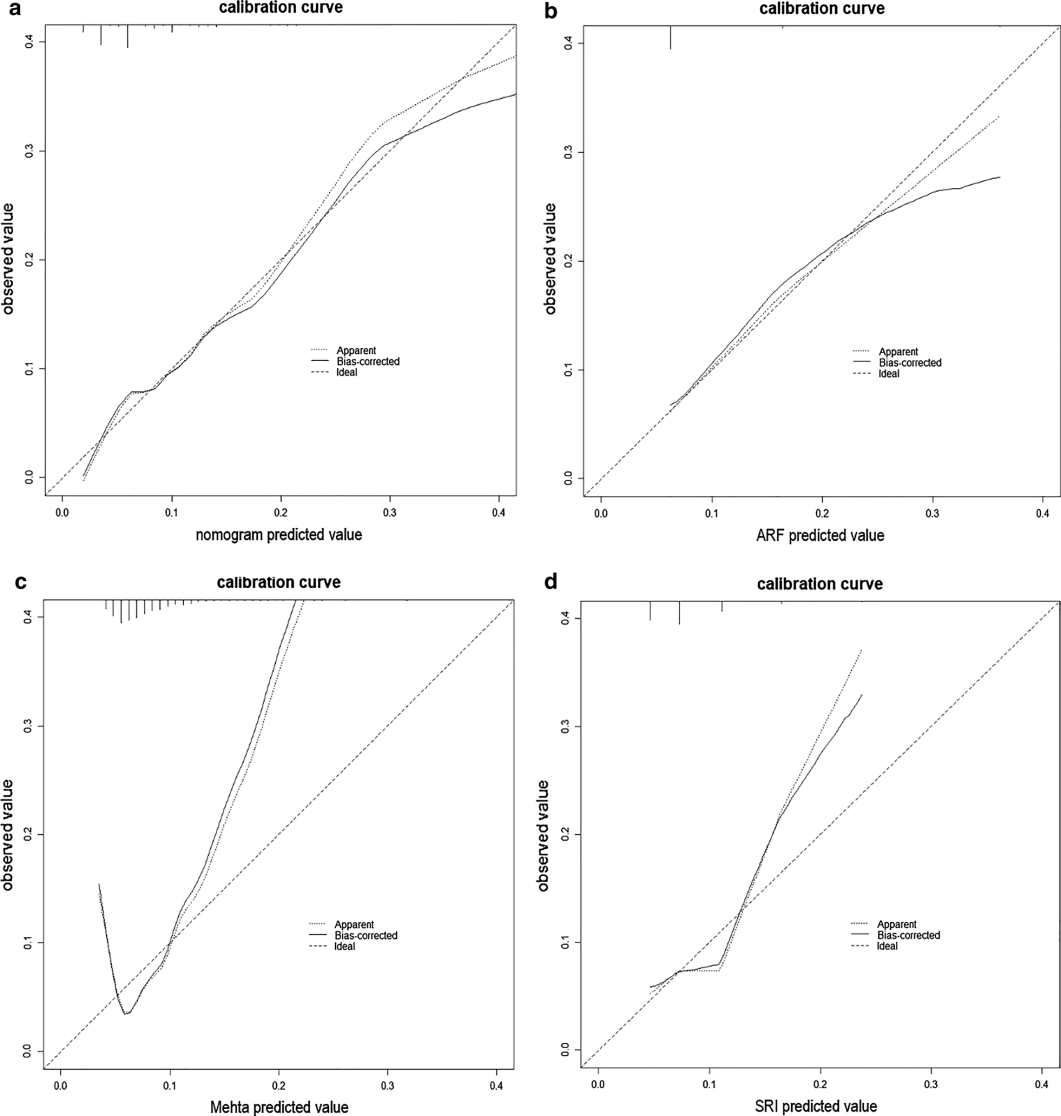
Calibration curves of 4 models in derivation cohort. **a** The novel nomogram model. **b** The Cleveland ARF model. **c** The Mehta model. **d** The SRI model

The calibration plots of the novel nomogram and the other three models indicated that the novel nomogram presented better performance in both derivation and validation cohorts (Fig. 2), especially for the patients whose expected AKI probabilities were below 40%.

**Fig. 3:**
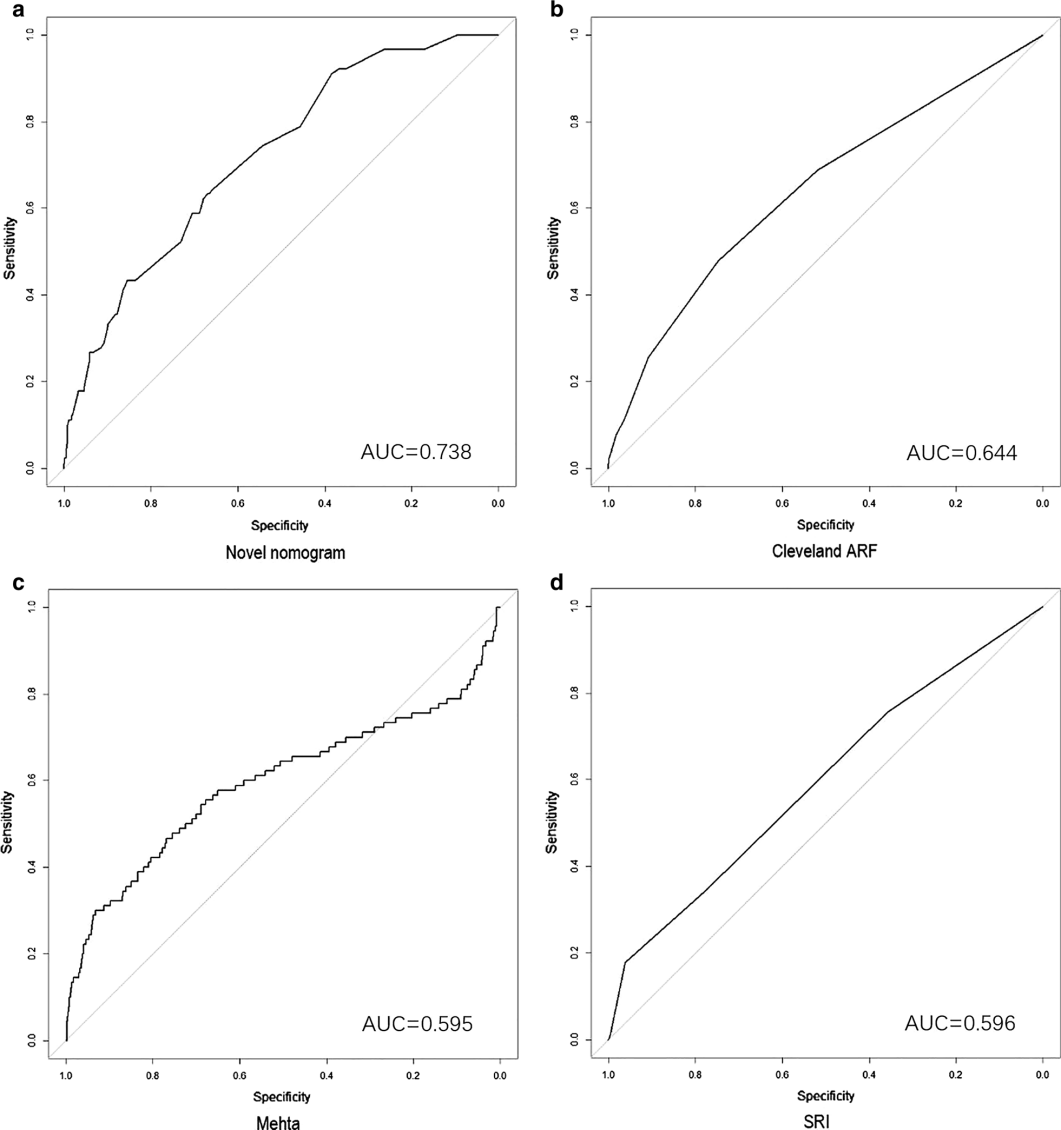
ROC curves of 4 models in derivation cohort. **a** The novel nomogram model. **b** The Cleveland ARF model. **c** The Mehta model. **d** The SRI model

**Fig. 4:**
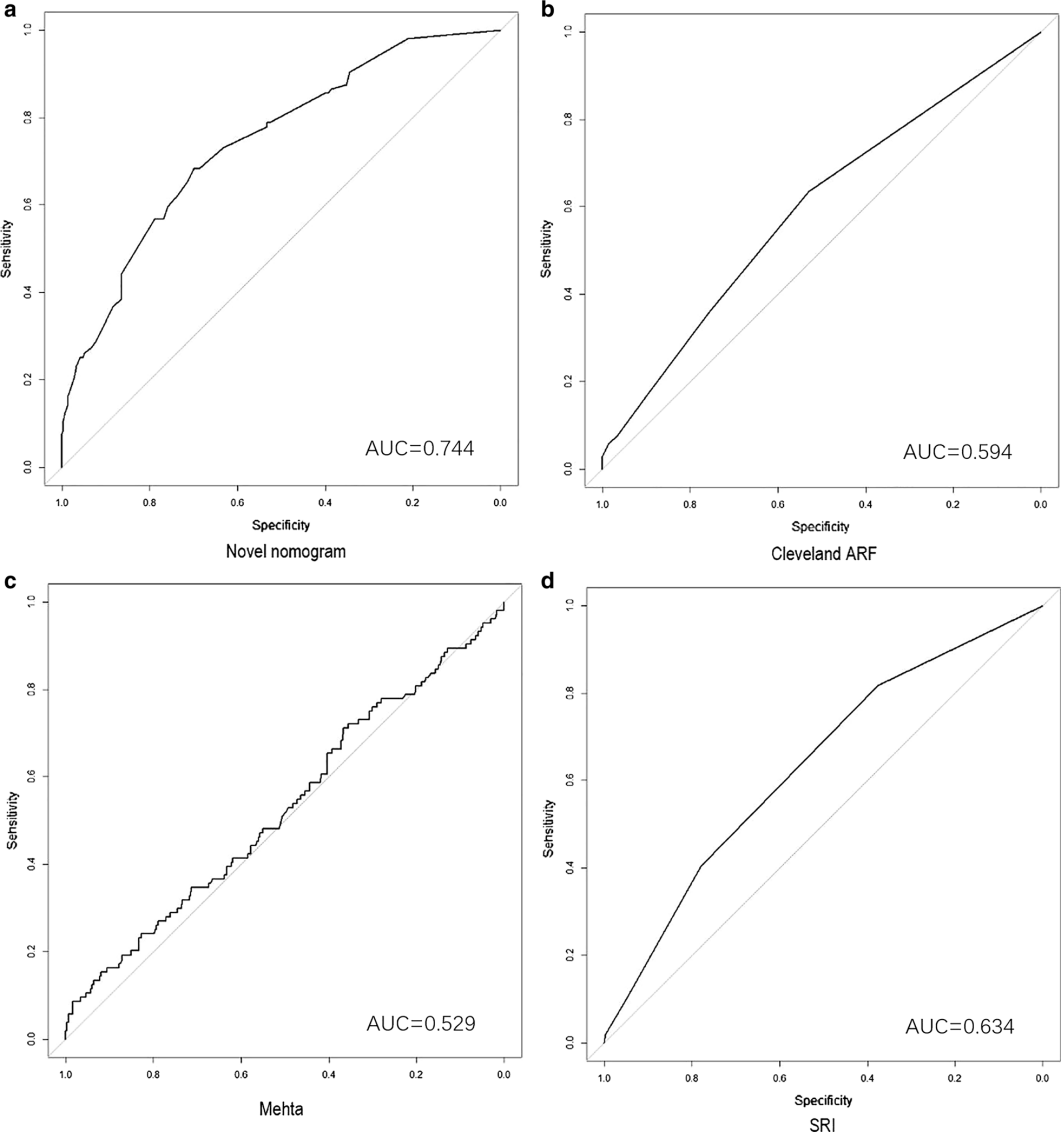
ROC curves of 4 models in validation cohort. **a** The novel nomogram model. **b** The Cleveland ARF model. **c** The Mehta model. **d** The SRI model

The AUCs of the novel nomogram were 0.738 and 0.744 in derivation and validation cohorts. Whereas, in derivation and validation cohorts, the AUCs of Cleveland score were 0.644 and 0.594, 0.595 and 0.529 for Mehta score, 0.596 and 0.634 for SRI score. The ROC curves were presented in Figs. 3 and 4.

**Fig. 5:**
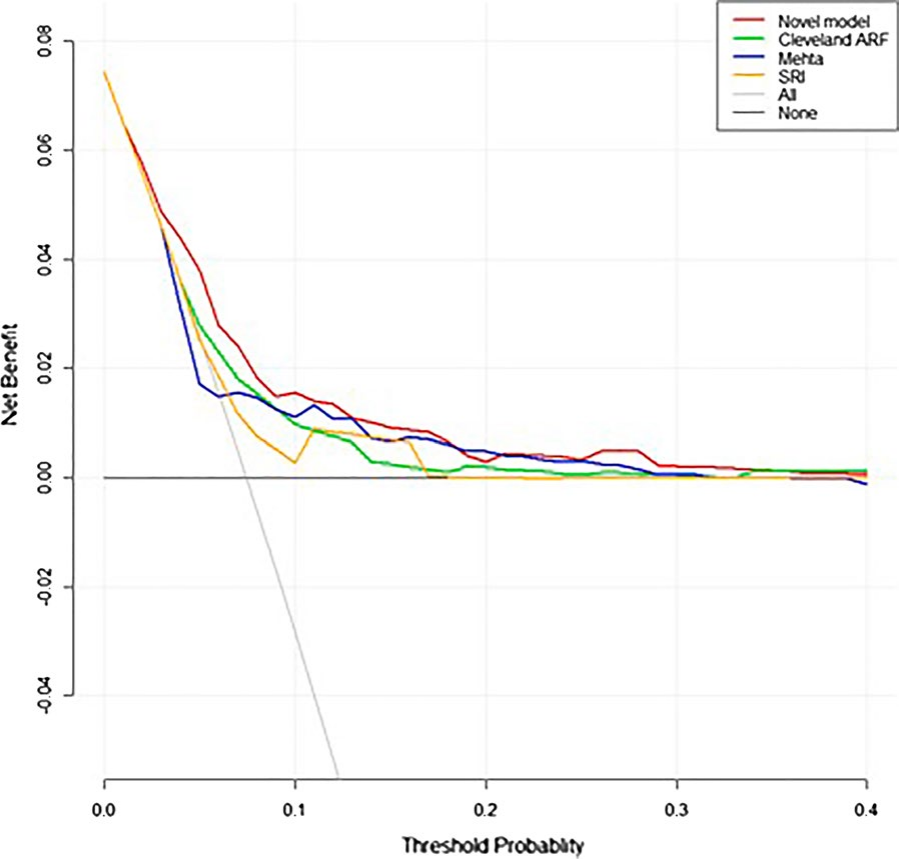
DCA curves of 4 models in derivation cohort

**Fig. 6:**
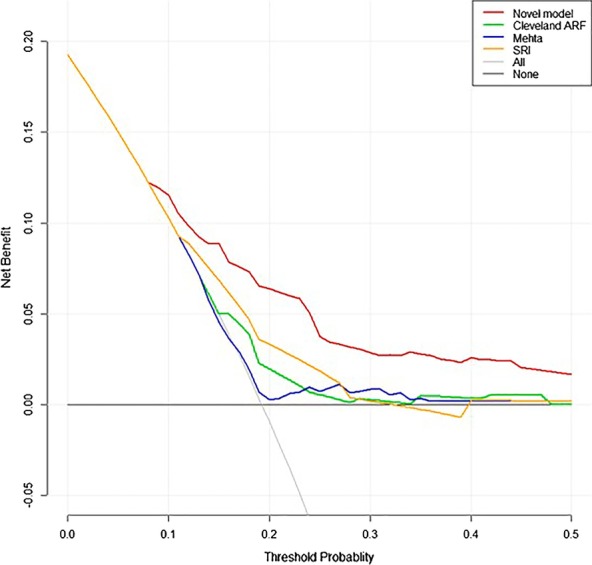
DCA curves of 4 models in validation cohort

The DCA curves demonstrated that the novel nomogram also displayed a higher clinical net benefit than the other three models both in derivation and validation cohorts (Figs. 5 and 6).

## Discussion

In this multicenter retrospective study, we selected 21 risk factors (Table 1) which were closely associated with prognosis of cardiac surgery and had been incorporated in some outstanding predictive models [3, 12]. We developed and validated a novel nomogram model based on 7 predictors to predict postoperative AKI in isolated CABG patients whose LVEFs were lower than 50%. These 7 significant predictors were female, SCr > 2 mg/dl, LVEF< 35%, previous MI, hypertension, CPB used and perioperative transfusion. Similarly, these 7 selected variables had been proved closely related to renal failure and incorporated in many other famous models [8–10]. Unlike the SRI model [10], for the sake of convenience, the novel nomogram included serum creatine (SCr) level instead of estimated glomerular filtration rate (eGFR) to exhibit renal function. As we aimed to construct a bedside predictive tool, SCr could be fast accessed and more suitable for bedside use than eGFR though the latter seemed more sensitive. Furthermore, the SCr performed well in our model and was also used in some other robust AKI prediction models [8, 9].

There were some differences between validation and derivation cohorts on demographic and operative risk factors. Given the heterogeneity of different cardiac centers, some predictors, like CPB used which was mostly determined by the operator, varied significantly between derivation and validation cohorts. CPB was used in 648/1208 (53.6%) cases in our center whereas only 70/540 (13%) cases were on-pump CABG in the validation cohort. A Laurie Shroyer et al. [13] conducted a randomized controlled trial (18 centers, *n* = 2203) which suggested that off-pump CABG led to lower rates of 5-year survival and higher rates of cardiovascular events and repeat CABG. Similarly, Christian H Møller et al. [14] reported a systemic review (86 trials and 10,716 participants) indicating a better long-term survival of on-pump CABG. Although CPB was related to a higher rate of postoperative AKI, it would not alter long-term kidney function compared with off-pump CABG [15]. Therefore, our center preferred an on-pump CABG for its better outcome. Except for CPB used and perioperative transfusion, rates of other predictors incorporated in the novel nomogram did not differ between groups (Table. 2). However, in terms of factors excluded from the model, compared with multi-center data, the baseline data of the patients in our center was characterized by higher incidences of extracardiac arteriopathy, previous cardiac surgery, NYHA III or IV, CCS 4 and non-elective surgery (Table 1). These characteristics indicated greater severity of the primary cardiovascular disease. As while as the multi-center population showed older age, more chronic kidney diseases, more diabetes and PCIs (Table 1). Suggesting that the validation group had more concomitant non-cardiac diseases with milder cardiovascular diseases, and the corresponding incidence of AKI was also higher. The derivation cohort and the validation cohort were retrieved from different populations. The derivation group used single-center data, while the validation group used multi-center data. The difference of baseline data between the derivation and the validation group did not affect the prediction ability of the model itself. Moreover, this model had better predictive performance in the validation group (multi-center and nationwide) than the derivation group with respect to AUC value and clinical net benefit, which further demonstrated the universality of this model.

The rate of AKI was higher in the nationwide multi-center validation cohort (19.3%) than the single-center derivation group (7.5%). The difference might stem from the gaps of therapeutic skills. In spite of this, the novel nomogram performed even better in validation group (AUC = 0.744 versus 0.738 in derivation group) whose AKI rate was significantly higher. The result suggested that the novel nomogram might be more suitable to the population with higher expected AKI rate. The calibration curve (Fig. 2 a) showed that the model might present the most precise prediction when the expected AKI rate was 10–30%.

Although LVEF< 50% and heart failure are two different concepts, they are closely related. We selected cases whose LVEFs were lower than 50% for reasons as follow: 1) Impaired LVEF is a crucial precursor of HF. If a patient without heart failure has a LVEF less than 50%, he or she can easily develop heart failure without timely intervention. 2) LVEF< 50% indicates a poorer prognosis compared with HFpEF (heart failure with preserved LVEF, LVEF≥50%) [16]. 3) The LVEF value measurement is relatively objective and has a good quantitative effect, which could facilitate the selection of patients with higher homogeneity (narrow case-mix) and improve the predictive ability of the derived model. Whereas, the diagnosis of heart failure is based on clinical symptoms and signs, which were usually determined by subjective judgment of clinicians. Patients with impaired EF usually have systolic or diastolic left ventricular dysfunction [16]. Therefore, avoiding AKI should be of greater importance in light of cardiac dysfunction.

Over the past decade, a rapid increasing number of impaired LVEF or heart failure patients have undergone CABG. Chinese CABG Registry [17] and a survey from India [18] have reported that about 10% of all the CABG patients had LVEFs which were less than 50%. AKI affects 12–48% of patients undergoing CABG [6], it contributes to increased mortality and decreased quality of life [19–21], especially for those with heart failure or impaired LVEF. Numerous prognostic risk models for AKI requiring renal replacement after cardiac surgery have been introduced into current practices. Unlike other models, we selected AKI (not AKI requiring renal replacement) as our predicting target because of its higher incidence rate and explicit criteria, whereas the dialysis events are rare (1–2%) [22]. Given the more concise and convenient diagnosis of AKI (not renal replacement), earlier prediction and recognition of the mild to moderate AKI seemed profound in postoperative management.

In spite of extensive application of some available predicting models [8–10], the benefits of utilizing these risk models might be very limited due to their poor predictive performance in those isolated CABG patients with impaired LVEF as we displayed in our results. Consequently, an accurate and convenient prediction model for AKI after isolated CABG would be invaluable for clinical practices.

The novel nomogram prediction model had demonstrated better predictive performance in calibration, discrimination and net benefit than the other three models. We thought it might be attributed to reasons as followed: (1) Specific for milder AKI. As we mentioned above, milder AKI is more frequent and contributes to several outcomes, making it more valuable for prediction. The other three robust models are for severe AKI requiring dialysis, less suitable for milder AKI. (2) Updated definition of AKI. With the progressive research, the understanding of AKI is also differing from the previous studies. This novel model was according to the latest guidelines for AKI. (3) New data. With the improvement of surgical techniques and perioperative management in recent years, the incidence of surgical complications is gradually reduced. The current widely used models were mostly based on old data of decades ago, which could be outdated. (4) Impaired LVEF. Our model was particularly for isolated CABG patients with impaired LVEF. However, nearly all the existing models were for general cardiac surgery patients, not specific for impaired LVEF. (5) Population heterogeneity. Wessler et al. [23] conducted a research suggesting that clinical prediction models may have variable performance across various databases, especially in many areas of Eastern Europe, Asia, Central America, South America, and Africa where much remains unknown. This is especially important considering the many regional differences in etiology, access to technologies, care systems, and guidelines. Other models were mostly developed on western populations and might be less applicable in Asian or Chinese populations.

Wessler et al. [23] reported a systemic review on 46 different clinical prediction models for cardiac surgery indicating that most models had poorer performance when tested in external validation cohorts and there was a median percentage change in discrimination of − 27.1% (interquartile range, − 49.4%–-5.7%). They thought this was likely the result of differences between the derivation population and the validation population where those models were developed on more highly selected (narrow case-mix) cohorts than they were testing on. Interestingly, in current study, our results demonstrated that the novel nomogram model had improved discrimination (AUC = 7.44 vs 7.38) and higher net benefit (Figs. 5 and 6) in the external validation. We thought the better performance in external validation may be attributable to the less heterogeneity between derivation and validation populations. Concretely, the cases in derivation and validation cohorts were both highly selected with respect to procedure type (isolated CABG without concomitant cardiac or aortic surgical operations), therapeutic era (same period) and technological levels (all the cases were selected from major cardiac centers with similar technological levels in China). In view that the cases of the validation cohort were national-widely recruited from different major cardiac centers and the testing outcome was promising, the novel nomogram model could be more suitable for Chinese or Asian populations than existing available models.

### Advantages of this novel model

This novel model only incorporated 7 variables which could be conveniently and noninvasively accessed in daily clinical practice. Therefore, it would be easier to be utilized and worthy for clinical popularizing.

### Limitations

Our findings should be interpreted in the context of the study’s limitations. First, our sample size of 1208 patients in derivation cohort and 540 patients in validation cohort was relatively small compared with other robust and widely used models. Second, our derivation cohort was based on a single-center database. Due to some missing data in China-HFSR which was a multi-center database, the model could be hardly derived from the multi-center cohort. Third, for a variety of reasons, data of some patients’ urine tests were not available, therefore factors like proteinuria which was reported related to adverse renal outcomes after CABG [24] failed to be included as potential predictors. Prospective large-scale studies with more detailed data are needed to further verify or update the current results.

## Conclusion

The novel nomogram model demonstrated better predictive performance than some widely used models and would be a reliable predictive tool for AKI after isolated CABG.

## Supplementary information

**Additional file 1:** Supplemental Appendices.

## Data Availability

The datasets used and/or analysed during the current study are available from the corresponding author on reasonable request.

## Abbreviations

AKI: Acute kidney injury
AUC: Area under the receiver operating characteristic curve
CABG: Coronary artery bypass graft
CAD: Coronary artery disease
CCS: Canadian cardiovascular society
China-HFSR: China heart failure surgery registry
CPB: Cardiopulmonary bypass
CT: Computerized tomography
DCA: Decision curve analyses
eGFR: Estimated glomerular filtration rate
HF: Heart failure
HFpEF: Heart failure with preserved EF
LVEF: Left ventricular ejection fraction
MI: Myocardial infarction
NYHA: New York heart association
SCr: Serum creatinine
