# Supplementary material for: A novel nomogram to predict perioperative acute kidney injury following isolated coronary artery bypass grafting surgery with impaired left ventricular ejection fraction
Source: BMC Cardiovasc Disord. 2020 Dec 10;20:517. doi: 10.1186/s12872-020-01799-1 (PMC7731767; doi:10.1186/s12872-020-01799-1)
Supplement: Supplementary file 1 — Additional file 1: Supplemental Appendices. [file 12872_2020_1799_MOESM1_ESM.docx]

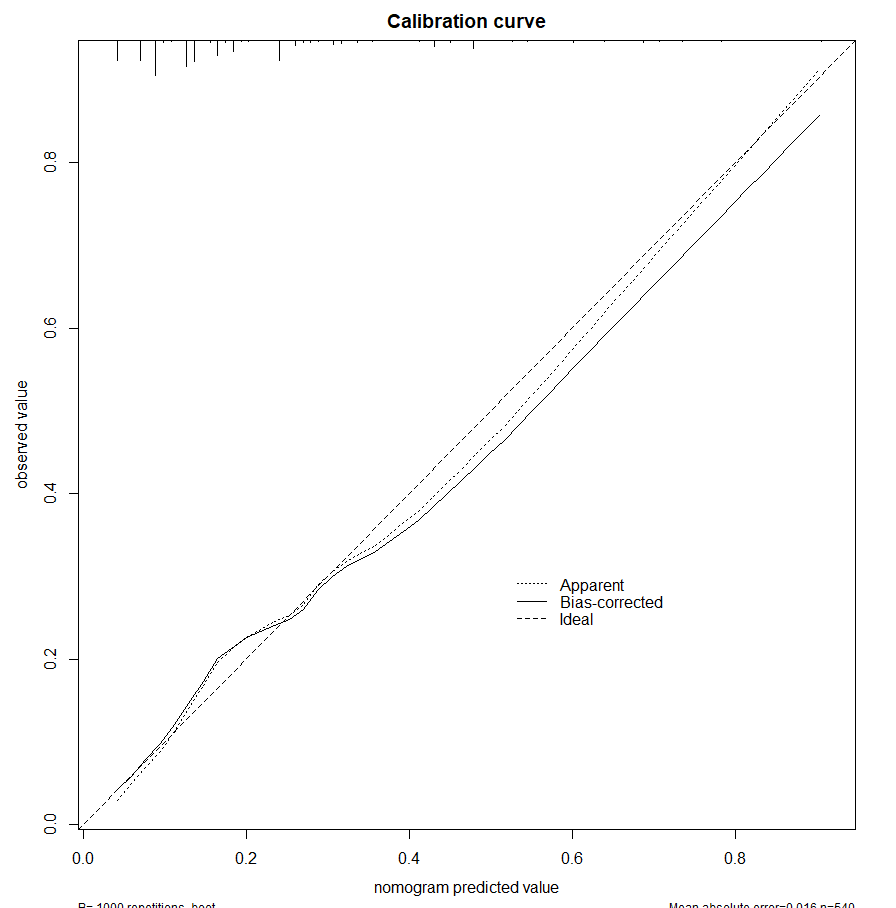


Additional file 1: Figure1. Calibration plot of the novel nomogram in validation cohort (n = 540)


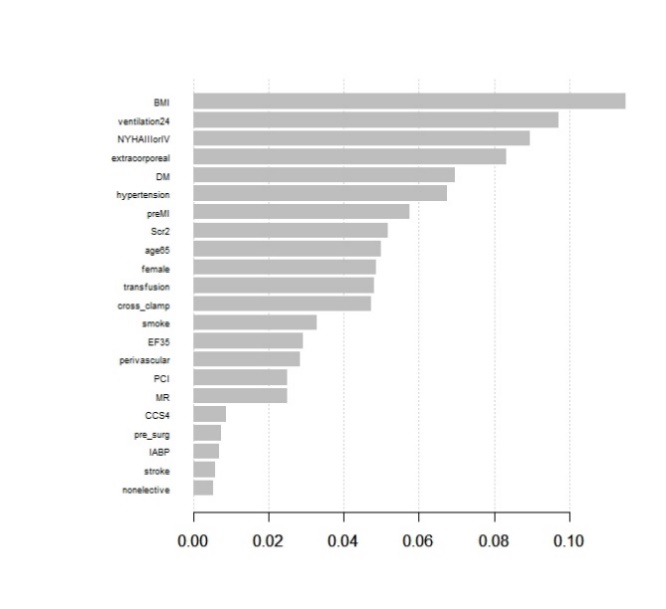


Additional file 1: Figure 2. Importance matrix plot of the machine learning model


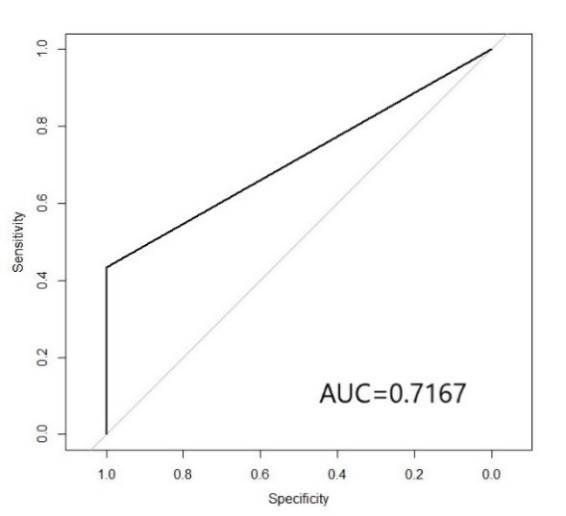


Additional file 1: Figure 3. ROC curve of the machine learning model
